# Internal fixation and muscle pedicle bone grafting in femoral neck fractures

**DOI:** 10.4103/0019-5413.38579

**Published:** 2008

**Authors:** AK Gupta, Sanjai Rastogi, R Nath

**Affiliations:** Department of Orthopedics, GSVM Medical College, Kanpur University, Kanpur - 208 002, India

**Keywords:** Femoral neck fracture, open reduction, internal fixation, muscle pedicle bone grafting, quadratus femoris muscle pedicle bone grafting

## Abstract

**Background:**

The treatment of displaced intracapsular femoral neck fracture is still an unsolved problem. Non-union and avascular necrosis are the two main complications of this fracture, especially if patient presents late. Muscle pedicle bone grafting has been advocated to provide additional blood supply. We present analysis of our 32 cases of displaced femoral neck fracture treated by internal fixation and quadratus femoris based muscle pedicle bone grafting.

**Materials and Methods:**

Open reduction and internal fixation with muscle pedicle grafting was done in 32 patients. The age of patients varied from 14-62 years (average age 45 years) with male to female ratio of 13:3. Twenty-nine fractures were more than three weeks old. All the cases were treated by Meyers' procedure. The fracture was internally fixed after open reduction and then a muscle pedicle graft was applied. It was supplemented by cancellous bone graft in seven cases. Fixation was done by parallel cancellous lag screws (*n* = 19), crossed Garden's screws (*n* = 7), parallel Asnis screws (*n* = 5) and Moore's pin (*n* = 1).Quadratus femoris muscle pedicle graft was used in 32 cases. In the initial 12 cases the graft was fixed with circumferential proline sutures, but later, to provide a secure fixation, the graft was fixed with a cancellous screw (*n* = 20). Postoperative full weight bearing was deferred to an average of 10 weeks.

**Results:**

Union was achieved in 26/29 (89.65%) cases which could be followed for an average period of 3.4 years, (2-8.5 years) with good functional results and had the ability to squat and sit cross-legged. Results were based on hip rating system given by Salvatti and Wilson. The results were excellent in 15 cases, good in four cases, fair in four cases and poor in six cases. Complications were avascular necrosis (*n* = 2), transient foot drop (*n* = 2), coxa-vara (*n* = 1) and temporary loss of scrotal sensation (*n* = 1).

**Conclusion:**

Muscle pedicle bone grafting with internal fixation is a viable treatment option in displaced femoral neck fractures with late presentation.

## INTRODUCTION

The treatment of femoral neck fracture (FNF) is still a matter of controversy even after so many recent advancements in Orthopedics. Nonunion (NU) and avascular necrosis (AVN) are the two main complications of this fracture. The rate of NU has been reduced by anatomical reduction and stable fixation of fractures but the incidence of AVN is evident.^1^

In 1962, the autogenous muscle pedicle graft based on the quadratus femoris muscle was used for the first time.^2^ Later, fresh autogenous cancellous iliac bone chips combined with muscle pedicle bone grafting have been reported to be good.^3^ There have been encouraging reports of Indian patients by using similar technique.^4^ We have treated 32 patients of displaced femoral neck fracture with late presentation by Meyers' technique. Three patients were lost to followup. So we are presenting analysis of the results of 29 patients treated by this method.

## MATERIALS AND METHODS

Thirty-two patients operated between 1984 and 1992 with a mean follow-up of 3.4 yrs. (range 2 years to 8.5 years) were included. All patients of displaced femoral neck fractures were treated by Meyers' procedure. Patients with femoral neck fracture with an inability to walk (due to reasons other than the femoral neck fracture), or with a life expectancy of less than five years, or with an inability to cooperate in the postoperative program were excluded from the study. All young patients with a displaced femoral neck fracture presenting late (more than 3 weeks old) or had unacceptabe closed reduction of a fresh fracture were included in the study.

Out of 32 patients, 26 were males and six were females. Their age ranged from 14 to 62 years (average age 45 years). 87.5% (*n* = 28) fractures were more than three weeks old at the time of operation. Road traffic accident was the commonest mode of trauma (*n* = 13), followed by fall from height (*n* = 11) and slip while walking (*n* = 8). The left side was involved in 21 patients and the right side in 11.Four patients presented with associated injuries (simple fracture both bone forearm, *n* = 2 and simple fracture calcaneum, *n* = 2). Majority (59.38%) of the fractures (19/32 cases) in our series were trans-cervical. The 13/32 cases (40.62%) were sub-capital. All the cases were Garden stage III, IV. All the patients were kept in below knee skin traction with Thomas knee splint while waiting for surgery.

### Surgical procedure

The patients were operated in prone position on a fracture table with adequate padding around perineal post and sacral support under suitable anesthesia. A close reduction under X-ray control was attempted; only minor adjustments were required under direct vision after the fracture site was exposed. The posterior Moore's approach was used in all cases. The quadratus femoris muscle was identified with its insertion on the quadratus tubercle at the intertrochanteric crest. The width of the graft was 1.5 cm and thickness was 1 cm.

An inverted “T” incision was made on the posterior part of the capsule and the fracture and posterior comminution were visualized directly. Final minor adjustments regarding anatomical reduction were done under direct vision. The defect in the posterior neck was packed with autogenous cancellous bone chips (harvested either from the posterior inferior iliac spine or the ipsilateral greater trochanter) prior to the placement of the screws. Fixation was done by parallel cancellous lag screws in 20 cases, crossed Garden's screw in seven cases, parallel Asnis screws in six cases and Moore's pin in one case. The length of the screws was confirmed under X-ray control.

**Figure 1:**
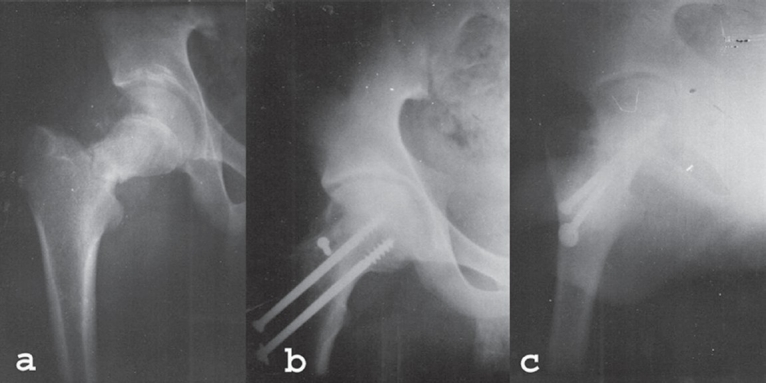
a,b,c: X-ray anteroposterior view of right hip shows femoral neck fracture (a), Two years followup X-rays AP and lateral views shows sound bony union and implant *in situ,* screw, used to fix muscle pedicle is visible (b and c)

The cephalic end of the graft was trimmed and a slot was made in the femoral head and posterior aspect of the neck across the fracture site. The graft was then placed into this slot and after impaction, it was firmly secured with a 4 mm cancellous lag screw (22 cases) [Figure 1] and circumferential proline suture in 12 cases. The wound was closed in layers over two suction drainage tubes. Mean time taken for surgery was 2 h 20 min and blood loss measured after closure was 250 ml to 350 ml.

The postoperative mode of immobilization depended upon the rigidity of the fixation and the age of the patient. Single hip spica was used in 26 patients and Thomas knee splint in six patients. During the period of immobilization, patients were encouraged to start active quadriceps exercises and non weight bearing exercises of hip and knee. The average period of immobilization in the postoperative phase was 10 weeks. Partial weight bearing was allowed gradually depending upon the status of union. Full weight bearing was allowed only after full osseous union, on an average of 7.5 months after the operation (5-9 months).

## RESULTS

**Figure 2:**
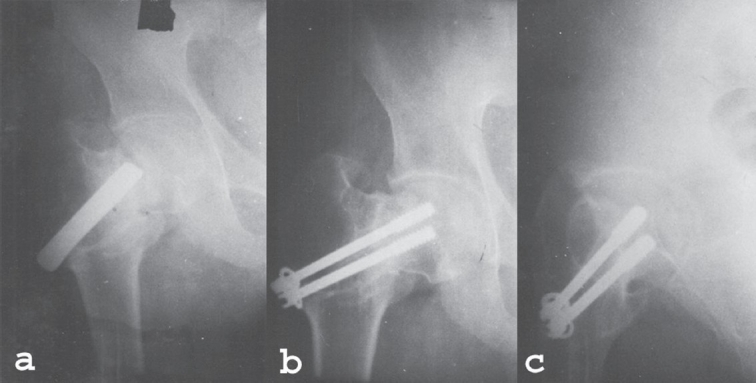
a,b,c: X-ray anteroposterior view showing internal fixation with non-union in a followup case (a), Three years followup X-ray showing sound union, implant *in situ,* coxa vara (b) and X-ray lateral view showing bony union (c)

The results were assessed in terms of osseous union. There was clinical as well as radiological union in 26 cases out 29 patients, which could be followed. Three patients were lost to follow-up. Osseous union and AVN were two major parameters to assess the results. We have followed the hip rating system given by Salvatti and Wilson^5^ for evaluation of hip function. The results were excellent in 15 cases, good in four cases, fair in four cases and poor in six cases. The important complications noted were AVN in 2/29 cases (6.89%), coxa-vara in 1/29 case (3.45%), transient foot drop in 2/32 cases (6.25%) and temporary loss of scrotal sensation in 1/32 case (3.12%). Transient foot drop and scrotal sensation recovered within six weeks time. Two patients, who developed AVN, were advised total hip arthroplasty but refused further treatment because of economic reasons. One patient who developed coxa vara [Figure 2] was functionally satisfied, hence refused further treatment.

## DISCUSSION

Anatomical reduction, impaction and rigid internal fixation are essential in treating femoral neck fractures. Muscle pedicle bone grafting has been advocated by many investigators along with rigid internal fixation to prevent NU and AVN of the femoral head. This provides an additional source of blood supply for the femoral head which may have been rendered ischemic by the fracture and also allows reduction and impaction of the fracture under direct vision.^3^,^4^,^6^

The severity of the comminution of the posterior cortex of the neck is observed quite frequently.^2^ The marked deficiency in the intramedullary bone of the femoral neck and head prevents rigid fixation of the fragments by any metallic implant. The muscle pedicle bone grafting provides some structural stability to the posterior aspect of the neck even when posterior cortex has been severely comminuted.

We have also used cancellous bone chips to fill up any gap at the fracture site before internal fixation is done. This increased the stability of the fracture reduction. We harvested cancelleous bone chips from either the posterior inferior iliac spine or from the greater trochanter.

Our results are quite comparable with the results of the Western series^3^ as well as Indian series [Table 1].^4^ These early results suggest that a marked reduction in the incidence of late segmental collapse and AVN of the femoral head was achieved by muscle pedicle bone grafting technique. We believe that this graft stimulates early and complete revascularization of the head of the femur following displaced fractures of the femoral neck by providing an additional source of vascular supply when the original one has been reduced by the fracture.

**Table 1:** Comparison of results with other series

Author's	Total No. of cases	Union %	Avascular necrosis
Meyers'	181	90	5%
Bakshi	56	75	
Our series	32	89.65	6.89%

We have used parallel cancellous lag screws in 19 cases, crossed garden screws in seven cases, parallel Asnis screws in five cases and Moore's pin in one case. Lag screws' fixation provides the best rigid fixation.^7^ Meyers' *et al.*^3^ and Baksi^4^ have preferred modified Hagie pins fixation in their studies.

Initially we fixed the muscle pedicle bone graft with circumferential prolene sutures (12 cases) but later on we switched over to 4 mm cancellous screw for better fixation. Graft dislodgement was not seen in any patient. It was easy to fix the graft with screw and fixation was better. Meyers' in his series also preferred graft fixation with screw while Baksi preferred circumferential silk suture for the fixation of graft.

The mean delay in surgery was five weeks. The various reasons for the delay included non-compliance of the patient for surgery, low socio-economic status of the patients, illiteracy, patients being unfit for the surgery. Hence this procedure is of special significance to those areas of the world where late reporting is not uncommon.

We agree with the concept of Meyers that posterior approach hardly damages further vascularity of the femoral head as the main lateral epiphyseal vessel lies in the superior part rather than in the posterior part of the capsule. We have not encountered any arterial bleeding during cutting the capsule posteriorly. This procedure requires no special equipment over and above that used for a standard internal fixation procedure and that prosthetic replacement can be easily carried out as a revision surgery in the failed cases.

## CONCLUSION

We conclude that muscle pedicle bone grafting with internal fixation is a very rewarding method of treatment of displaced fracture of femoral neck with late presentation.
